# Initial experience of benign upper gastrointestinal robotic-assisted surgery: first 200 cases and early postoperative outcomes

**DOI:** 10.1308/rcsann.2024.0093

**Published:** 2025-04-03

**Authors:** K Greene, EJ Nevins, T Akharaekpanya, S Bawa, L Horgan

**Affiliations:** Northumbria Healthcare NHS Foundation Trust, UK

**Keywords:** Robotic-assisted surgery, Minimally invasive surgery, Robotic outcomes

## Abstract

**Introduction:**

Robotic-assisted surgery is an alternative approach to minimally invasive surgery for benign upper gastrointestinal (UGI) conditions and abdominal wall hernia – its application in the United Kingdom is still in the initial phases in many National Health Service (NHS) trusts. We detail the experience of Northumbria Healthcare NHS Foundation Trust in implementing a robotic-assisted surgery service for benign UGI procedures and abdominal wall hernia repair.

**Methods:**

The robotic service for benign UGI was established in the trust in February 2022. All theatre staff received online and simulation training before working in the dedicated robotic surgery theatre. Operative timings, surgical outcome measures and patient outcomes including day-case rates were prospectively recorded and analysed to assess the impact of the introduction of this service.

**Results:**

Between February 2022 and June 2023, some 200 robotic-assisted procedures were performed: cholecystectomy (*n* = 103), hernia repair (*n* = 74), anti-reflux surgery (*n* = 9) and Heller's myotomy (*n* = 14). Median docking times were recorded: cholecystectomy, 9min (4–94min); hernia repair, 10min (4–50min); anti-reflux surgery, 19min (9–37min); and Heller's myotomy, 15min (6–26min). There were no intraoperative complications. Two patients returned to theatre for bile leak following cholecystectomy, presenting on day 2 and day 9 postoperatively.

**Discussion:**

Robotic-assisted benign UGI surgery can be safely performed in a day-case centre and does not impact day-case rates. There were no theatre delays because of prolonged docking times, even in the initial introductory period. There are higher costs associated with robotic-assisted surgery; however, with time and industry development, these are likely to improve.

## Introduction

By the end of the 20th century, laparoscopic surgery had become widely recognised as a superior method for minimally invasive surgery. More recently, robotic-assisted surgery has expanded across surgical specialties to offer an enhanced approach to minimally invasive surgery.^1–3^ Both laparoscopic and robotic-assisted surgery involve the use of small, ‘port’ incisions, a camera and surgical instruments to perform a procedure. Robotic-assisted surgery, performed through use of an operating console, has the additional benefits of three-dimensional imaging, articulated instruments and a tremor filter which can improve dexterity and optics. The application of robotic-assisted surgery for benign upper gastrointestinal (UGI) conditions and abdominal wall hernia is still relatively new in the United Kingdom (UK) with limited evidence to compare outcomes with laparoscopic surgery. There is literature from the United States, which suggests that although robotic-assisted surgery is associated with increased duration of surgery and cost of procedure, patient outcomes are improved.^4^

Benign UGI procedures include cholecystectomy, inguinal hernia repair, ventral hernia repair, fundoplication, paraesophageal hernia repair and Heller's myotomy, which can be performed using open or laparoscopic approaches. Robotic surgery can improve dexterity and enhance precision during minimally invasive surgery, and although some literature suggests that there are lower rates of complications and readmission in patients undergoing robotic cholecystectomy, larger studies have shown a reduction in 90-day readmission rates with robotic-assisted cholecystectomy.^4,5^

Robotic-assisted surgery is associated with higher costs than laparoscopic surgery; however, with the increasing application of robotic-assisted surgery worldwide, it is likely that we will see reductions in cost of equipment and increased availability for alternative suppliers.^6^

As access to surgical robots increases across the UK, we share our experience in establishing a benign UGI robotic service in Northumbria Healthcare NHS Foundation Trust. In 2020, the trust funded a new robotic surgery programme, which included two fully equipped DaVinci Xi robots for use in colorectal, UGI and gynaecology procedures, and two dedicated robotic Band 7 theatre nurses. All members of staff underwent online and simulation training together as a team. The aim of this publication is to outline how the team at our trust introduced a robotic-assisted service for benign UGI procedures, and to assess intraoperative timings and evaluate the safety profile of this new service by reviewing patient outcomes.

## Methods

The benign UGI robotic service was introduced in February 2022; prior to this, the trust had functioning colorectal and gynaecology robotic services from September 2021, which were led by an executive clinical group with engagement from managers and the wider theatre team. Two robotic Band 7 theatre nurses were appointed: one to work at each of the two sites where robotic-assisted procedures were performed, to ensure co-ordination of surgical services and to assist with training the surgical and nursing staff involved in the robotic cases. Northumbria is a multisite trust with elective laparoscopic benign UGI surgery offered at four hospitals within the trust. The robotic service for benign UGI was established at North Tyneside General Hospital where a laparoscopic theatre was modified to house the robotic equipment to industry standards.

Department-wide education and safety training was delivered to all members of the theatre team to ensure patient safety and to engage the team in the process of establishing a new service. All team members were given access to the robotic modular training simulator so the whole team could appreciate the potential benefits to patients and the operating surgeon – this experience helped to increase staff support towards the introduction of the robotic services. Safety in the application of this technology is paramount, and so all members of staff were required to complete online and bedside training prior to any involvement in a robotic-theatre case. All procedures were carried out in a dedicated robotic surgery theatre using the Da Vinci Xi robot.

A robotic surgery committee was established in the trust with both medical and nursing staff, and this committee helped to oversee the provision of robotic training and the utilisation of robotic theatres. In light of concerns regarding reduced efficiency in theatre during the initial phase of establishing a robotic benign UGI service, and the pressures of waiting lists for these procedures, two consultants were selected to begin operating with the robot with a phased programme of training and transition from laparoscopic to robotic-assisted in the department.

Two experienced consultant surgeons (LFH and SB) with advanced laparoscopic skills in benign UGI procedures were fully trained in the use of the DaVinci Xi robot using the intuitive training programme and proctoring. Once their training was complete, these two consultants worked in tandem for each case in the series: one surgeon on the console and the other surgeon as the bedside assistant. During the theatre list, surgeons would alternate roles for sequential cases, and this approach was continued for the first 200 cases to build an experience level that could facilitate in-house proctoring and mentoring of all consultants in the department who wish to transition to robotic practice.

Anaesthetic was provided by regular robotic-theatre anaesthetic consultants who were actively involved in the robotic team and were aware of the safety measures that may differ from standard laparoscopic cases.

From inception of the robotic-assisted surgery programme, the robotic surgery committee was focused on ensuring that surgical trainees had training, both theory-based and practical, in robotic-assisted surgery. Surgical trainees attended dedicated training sessions on bedside assisting during robotic cases, and this was followed by regular exposure in theatre as a bedside assistant to consolidate their learning. Trainees were also provided with access to the robotic simulator on both sites and encouraged to develop their skills. On a wider level, this training complemented the concurrent role of the Newcastle Surgical Training Centre robotic training programme – Utilising the Intuitive Da Vinci System – for Northern Deanery surgical trainees, which allowed trainees access to both simulator and cadaveric operating. This process was certified to ensure that trainee progress is recorded.

Patients were selected from those referred to UGI outpatient clinics. Patients were informed that robot-assisted surgery was a newly established service and were offered a robotic-assisted or laparoscopic approach. Patients were counselled on the risks and benefits of the procedure in clinic and information leaflets were provided.

At the beginning of every robotic surgery list, an extended team brief was held in which all members of the team were introduced, the cases and equipment were discussed and each person was assigned an individual role. Particular attention was paid to individual roles during an emergency, during which un-docking of the robot would be required, and these roles were clearly written on a board on the wall that was available for reference throughout the list. At the end of every list, a debrief was held and every member of the team was offered the opportunity to highlight anything that went well, any challenges and any learning needs from the day.

A database was created to record outcomes for each case. It included patient demographics, procedural information and postoperative outcomes, which were recorded at 30 days using electronic records from the emergency department, surgical admissions unit and general practitioner regional primary care records. The team was conscious that one of the main concerns surrounding robotic surgery was time-efficiency and so specific timings were recorded prospectively: total operating room (OR) time – the time from patient arrival in the anaesthetic room, to patient extubation; docking time – the time between the first skin incision and the time when the operating surgeon takes control of the robotic console; and console time – the time between the first and last operative movement on the operative console.

The majority of patients who underwent cholecystectomy and hernia repair surgery did not have surgical follow-up, as is routine for our day-case UGI practice. The other patients were followed up at the consultant's discretion. Any complications of surgery were discussed at the monthly departmental morbidity and mortality meeting, as is the case with laparoscopic and open cases.

## Results

### Demographics

The first 200 cases in our series were performed between February 2022 to June 2023 across four operating list sessions per week on average. During this time, procedures performed included cholecystectomy (*n* = 103), hernia repair (*n* = 74), anti-reflux surgery (*n* = 9) and Heller's myotomy (*n* = 14). In addition to the cholecystectomies, there were four Common Bile Duct (CBD) explorations, two repair of cholecystoduodenal fistulae and one on-table cholangiogram performed. Of the hernia repairs, nine were bilateral procedures.

### Timings

The age of patients ranged from 18 to 94 years (mean 56). American Society of Anesthesiologists (ASA) grades ranged from 1 to 3 (1, 53 patients; 2, 121 patients; 3, 26 patients). The sex distribution of patients was evenly split with 96 female and 104 male patients. Some 55% of anti-reflux procedures were revision procedures.

**Figure 1:**
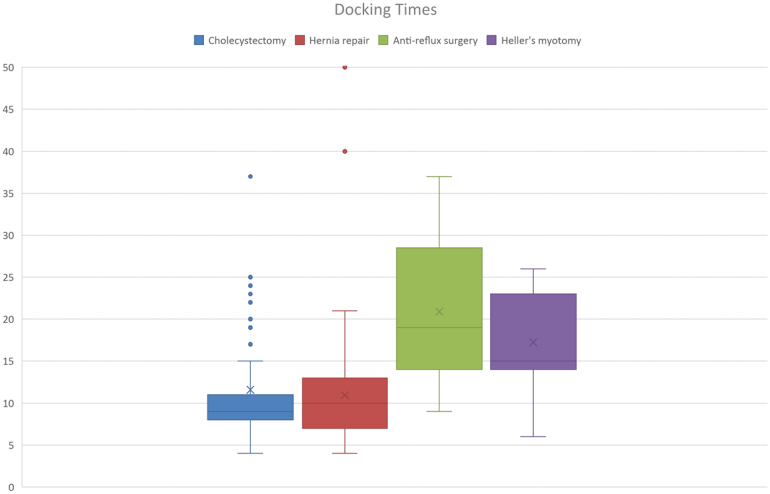
Box plot demonstrating docking time for each operation type with median value and interquartile ranges denoted

**Figure 2:**
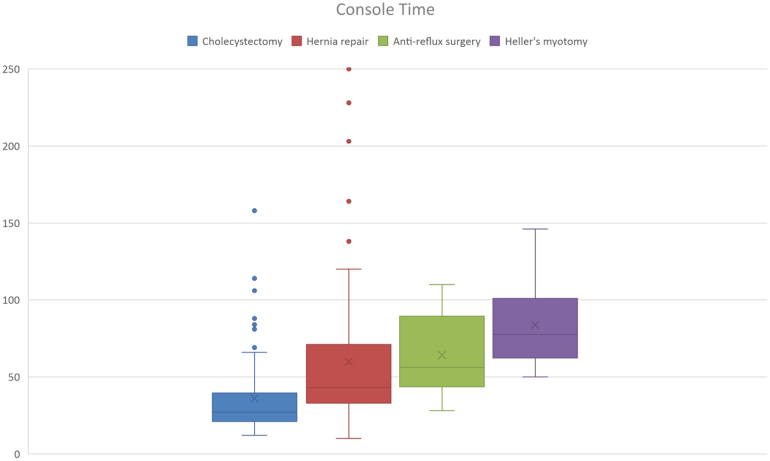
Box plot demonstrating console time for each operation type with median value and interquartile ranges denoted

**Figure 3:**
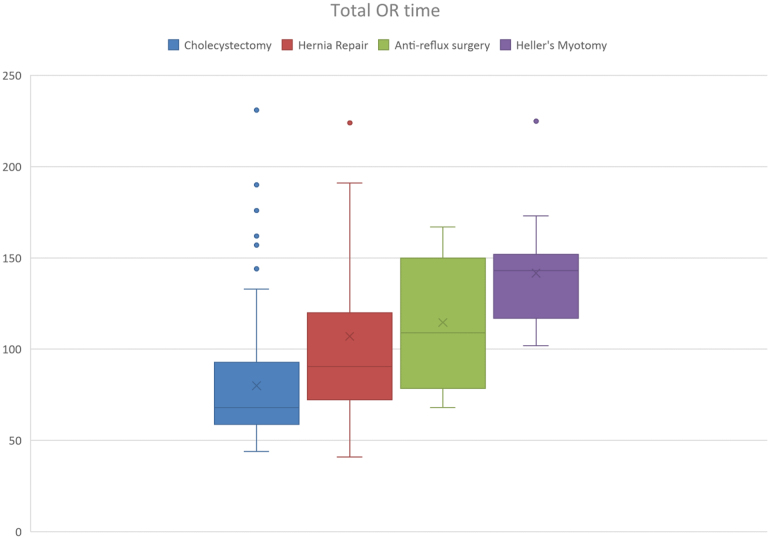
Box plot demonstrating operative time for each operation type with median value and interquartile ranges denoted.OR = operating room

One of the main concerns about robotic-assisted surgery is a reduction in theatre efficiency; robot docking times were therefore carefully recorded in addition to console time and total time in the operating theatre. These measurements were reviewed separately for each type of procedure. Figures 1–3 demonstrate the dockings times, console time and total OR time for each prodcedure type. These figures demonstrate that although there were some outliers, the docking times were comparable across each procedure. Console times were also comparable for each procedure type, taking the complexity of some of the cases into account.

**Figure 4:**
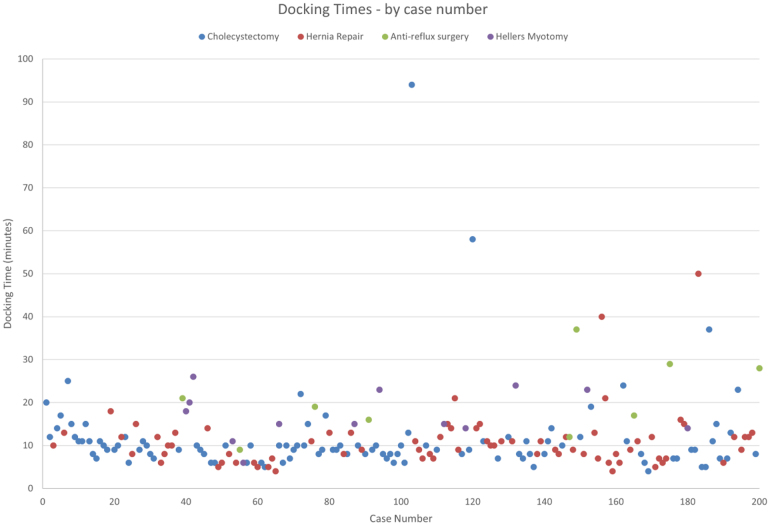
Graph demonstrating docking time by case number (1–200)

Robot docking time for cholecystectomy ranged from 4 to 94min (median 9min). Extensive adhesiolysis was required prior to completing docking in the case where docking time was 94min. Console time for cholecystectomy ranged from 12 to 160min (median 27min). In the four cases where operative time was >100min, further procedures were required (three CBD exploration, one adhesiolysis). Total OR time ranged from 44 to 231min (median 68min). Once again, the outliers for total OR time were those cases in which CBD exploration or extensive adhesiolysis was required (see Figure 4).

Hernia repairs performed included inguinal hernia repair (*n* = 41), umbilical hernia (*n* = 18), epigastric (*n* = 3), incisional hernia (*n* = 9), parastomal hernia (*n* = 1) and Spigelian hernia repair (*n* = 2). Robot docking time for hernia repair ranged from 4 to 50min (median 10min). Console time for hernia repair ranged from 10 to 250min (median 43min). Ten cases lasted >90min (console time), and these were either complex incisional herniae or bilateral repairs. Total OR time ranged from 41 to 318min (median 90min).

In all cases of anti-reflux procedures, a Nissen fundoplication or re-do Nissen fundoplication was performed. Robot docking time for anti-reflux surgery (Nissen fundoplication) was 9–37min (median 19min). Console time for anti-reflux surgery ranged from 28 to 110min (median 56min) and total OR time ranged from 68 to 167min (median 109min).

For all Heller's myotomy procedures, a 180-degree anterior fundoplication was also performed as part of the procedure as is standard in the trust. Robot docking time for Heller's myotomy ranged from 6 to 26min (median 15min). Console time and total OR time were 50–146min (median 77min) and 102–225min (median 143min), respectively.

The average number of cases per 4-h theatre session was two.

### Surgical outcome measures and complications

Blood loss for every procedure was recorded as a marker of surgical outcome for the patient and a relative patient safety indicator. There were no cases in which blood loss was >100ml.

All cases (100%) were completed robotically without the need for conversion to a laparoscopic or open approach. There were no intraoperative complications recorded across the 200 cases.

Postoperative complications within 30 days of surgery were recorded and discussed at the local morbidity and mortality meeting. Complications included seven surgical site infections, which were managed with antibiotics, four cases of urinary retention and one chest infection. In the cohort who underwent cholecystectomy, there were two bile leaks and one patient with a retained gallstone, which was not amenable to extraction during CBD exploration and passed spontaneously postoperatively. In the hernia repair group, three patients developed haematomas, which were managed conservatively and did not require readmission; one patient had a recurrence 4 months postoperatively. There were no major complications noted in the anti-reflux or Heller's myotomy group who reported good postoperative outcomes in resolution of symptoms and weight gain in the Heller's myotomy group. An in-depth review of outcomes for the Heller's myotomy cohort was completed.^7^

### Day-case rates and 30-day readmission/reoperation data

For each case, length of stay was recorded. Overall, 78% of cases were performed as day-case surgery. Forty-one cases were not day-case procedures and length of stay for these patients ranged from 1 to 49 days. Of these 41 patients, only 5 were due to complications (4 patients with urinary retention and 1 bile leak). Although as a unit we aim to perform hernia, cholecystectomy, fundoplication and Heller's myotomy procedures as day cases, 36 cases were planned admissions. We have a thorough preoperative assessment programme that highlighted the need for planned admission in these cases owing to comorbidity or social circumstances.

Some 2% of patients were readmitted within 30 days of their procedure. Two patients required reoperation within 30 days for a bile leak following cholecystectomy and CBD exploration; these were on the second and ninth postoperative day. There were no mortalities within 30 days of the procedures for the 200 cases. Because there were only two complications requiring operative management, it is feasible to perform robotic procedures in day-case centres. In the cases of complications in which patients required reoperation, this was a delayed presentation.

## Discussion

Robotic surgery has multiple applications in benign UGI and general surgery, as demonstrated by this series. In addition to routine cases, there is scope to do more advanced surgery where necessary, such as extensive adhesiolysis and CBD exploration using a robot-assisted approach. In the hands of an experienced laparoscopic surgeon, as the literature has demonstrated, robotic-assisted surgery has benefits such as improved optics, instrument articulation and instrument stability.^5,8^ This is critical so that patient safety and quality of care are not jeopardised by the implementation of a new technology.

One of the main concerns with robotic surgery is the additional time required both in the set-up of the robot and the additional time taken for surgeons to complete the procedure. Although there is undoubtedly a learning curve in the acquisition of a new skill and this can result in cases taking longer, our experience was that in the case of surgeons with an established laparoscopic practice, this was quickly overcome. Thorough training of theatre staff ensured their familiarity with the robot equipment and as such, there was no significant learning curve reflected in docking times for each case. With a mean docking time of 12min across the 200 cases, this did not pose significant delays to cases starting in theatre. Theatre staff were engaged in the process of recording their timings in theatre and this motivated them to ensure that the set-up of equipment was done efficiently.

We endorse two-consultant operating lists in the establishment of robotic surgery practice and highlight the additional safety feature of having two consultants present in the initial phase of implementing robotic surgery following the proctoring phase of training. We are continuing the progression of the robotic training programme in our unit and are now training the second phase of UGI surgical consultants. We plan on widening the cases that we perform on the robotic platform and hope in the future this will include robotic-assisted bariatric surgery and possibly some emergency general surgery procedures.

Utilising two consultant surgeons in the same list increases the cost per case; however, the trust took a stance to ensure that patient safety was maintained in preference to cost saving, and in an attempt to reduce errors and negate complications during the learning curve phase, they opted to simultaneously train two consultant surgeons. The investment in training these surgeons simultaneously has allowed their skills to be utilised in training other consultants in the unit.

The majority of cases were performed as day cases and our rates of complications and readmission were comparable with laparoscopic surgery. This highlights that robotic surgery, even in the initial 200 cases in our trust, did not lead to adverse patient outcomes or additional pressure on surgical services. Patients who attended outpatient clinics for follow-up reported positive experiences on the whole.

### Study limitations

Recent literature has identified that costs associated with robotic-assisted cholecystectomy are higher than laparoscopic cholecystectomy. One such study has identified a cost disparity of $5,500 per case; however, there has been no further clarity on how factors such as surgeon, hospital robotic-assisted surgery volume and operating time influenced this cost.^9^ We recognise that there are significant initial start-up costs associated with the purchase of the robotic system; however, we can demonstrate economical utilisation of the robotic system across the general surgery and gynaecology departments in our trust. The authors recognise that a limitation of the current study is that we have not performed an in-depth cost analysis of the introduction of this programme. Although, in the initial phase, robotic surgery is likely to be more expensive, with increasing usage of robotic surgery, staff familiarity and robotic manufacturer competition, barriers with regards to cost will be overcome. The authors believe that robotic surgery has an ongoing role in both complex and routine benign UGI surgery.

## Conclusion

Although there is a significant learning curve and financial cost associated with establishing a benign UGI robotic service, it offers enhanced precision and dexterity, which can be invaluable in more complex cases. Robotic-assisted surgery did not adversely affect theatre efficiency through delays secondary to docking or prolonged operative time. The introduction of robotic-assisted surgery did not have an impact on day-case rates. Robotic-assisted surgery is comparable to laparoscopic surgery for benign UGI procedures in terms of patient outcomes and day-case rates; however, it is associated with significant equipment costs and requires specific training of all team members to ensure patient safety. The experience and patient outcomes associated with establishing the robotic-assisted surgery for benign UGI cases has been encouraging. We hope that with ongoing industry development, this innovative approach to minimally invasive surgery will become more attainable and we will see wider application of robotic-assisted surgery in this field.
